# Identification and genetic analysis of Kadipiro virus isolated in Shandong province, China

**DOI:** 10.1186/s12985-018-0966-y

**Published:** 2018-04-06

**Authors:** Weijia Zhang, Fan Li, Aiguo Liu, Xiaojuan Lin, Shihong Fu, Jingdong Song, Guifang Liu, Nan Shao, Zexin Tao, Qianying Wang, Ying He, Wenwen Lei, Guodong Liang, Aiqiang Xu, Li Zhao, Huanyu Wang

**Affiliations:** 1 School of Public Health, Shandong University, Jinan, 250012 People’s Republic of China; 2 State Key Laboratory of Infectious Disease Prevention and Control, Chinese Center for Disease Control and Prevention, Beijing, People’s Republic of China; 3 Department of Viral Encephalitis, National Institute for Viral Disease Control and Prevention, Chinese Center for Disease Control and Prevention, Beijing, 102206 People’s Republic of China; 4 Institute for Immunization Program, Center for Disease Control and Prevention of Dongying City, Dongying, 257091 People’s Republic of China; 5 Institute for Immunization Program, Shandong Center for Disease Control and Prevention, Jinan, 250014 People’s Republic of China; 6 Department of Academician Hong Tao, National Institute for Viral Disease Control and Prevention, Chinese Center for Disease Control and Prevention, Beijing, 100052 People’s Republic of China

**Keywords:** Kadipiro virus, Genetic analysis, Identification, Seadornavirus

## Abstract

**Background:**

Kadipiro virus (KDV) belongs to the Reoviridae family, which consists of segmented, non-enveloped, double-stranded RNA viruses. It has previously been isolated from *Culex, Anopheles, Armigeres* and *Aedes* mosquitoes in Indonesia and China. Here, we describe the isolation and characterization of SDKL1625 from *Anopheles sinensis* mosquitoes in Shandong province, China.

**Methods:**

In this study, we isolated Kadipiro virus in *Aedes albopictus* C6/36 cell culture and the complete genome sequencing was made by next generation sequencing.

**Results:**

We isolated and characterized a Kadipiro virus from *Anopheles sinensis* mosquitoes in 2016 in Shandong province, China. Nucleotide and amino acid homology analysis of SDKL1625 showed higher levels of sequence identity with QTM27331 (Odonata, China, 2016) than with JKT-7075 (*Culex fuscocephalus*, Indonesia, 1981). The SDKL1625 has 86–97% amino acid identity with the JKT-7075, 88–99% amino acid identity with the QTM27331. Among the 12 fragments, VP1, VP2, VP4, VP6, VP7, VP9 and VP12 showed high amino acid identity (> 90%) and VP5 showed the lowest identity (86% and 88%).

**Conclusions:**

This is the first identification of KDV from mosquito in China. Virus morphology and genome organization were also determined, which will further enrich our understanding of the molecular biological characteristics of KDV and seadornaviruses.

## Background

Kadipiro virus (KDV) is a species of the new 12-segmented RNA virus genus *Seadornavirus*, which was assigned to the Reoviridae family in the eighth and ninth report of the International Committee on the Taxonomy of Viruses [1, 2]. The *Seadornavirus* genus includes three species, Banna virus (BAV), Kadipiro virus (KDV) and Liao ning virus (LNV). New species have been isolated in recent years, including the Balaton [3] and Mangshi viruses [4].

BAV is the prototype species of the genus *Seadornavirus* and was first isolated in 1987 from patients with encephalitis in Xishuangbanna prefecture, Yunnan province, China [5]. To date, BAV has been isolated from mosquito species, humans with encephalitis, pigs, cattle and ticks in Southeast Asia, particularly China and Indonesia [6, 7]. BAV is an emerging pathogen that causes human viral encephalitis, exhibits rich genetic diversity and great differentiation potential, and does not exhibit a species barrier [8]. The discovery of LNV, a new virus strain obtained from mosquitoes in China, enriched the variety of the *Seadornavirus* genus. LNV was able to replicate not only in mosquito cell lines, but also in mammalian cell lines, and thus may be able to infect mammals [9]. Phylogenetic analysis show LNV might be an emerging virus that evolved rapidly and is widely distributed in the northern part of China [10, 11].

KDV was first isolated from *Culex fuscocephalus* in Java, Indonesia in 1981 by replication in the C6/36 cell line, grouped into the genus *Coltivirus* within the family Reoviridae as the enough genetic information could not be obtained to permit exact classification [7, 12]. Since then, KDV has also been isolated from *Culex tritaeniorhynchus*, *Anopheles sinensis* and *Armigeres subalbatus* in northwestern Yunnan province, China [13]. To date, only two full genome KDV sequences have been recorded in GenBank [14, 15]. Recently, a viral metagenomics study showed that a plasma sample of a febrile adult, who enrolled in a study of acute human immunodeficiency virus type 1 (HIV-1) infection in coastal Kenya, exhibited translated protein matches to KDV virus. This discovery indicated that the tropism of KDV includes humans [16].

Here, we have isolated a strain of KDV from *Anopheles sinensis* mosquitoes in Kenli county, Shandong province, China. We describe the isolation and characterization, as well as genomic analyses, of this virus and its phylogenetic relationship with members of *Seadornavirus*, which will further enrich our understanding of the molecular biological characteristics of KDV and seadornaviruses.

## Methods

### Mosquito collection

Mosquito samples were collected on September 7, 2016 in residential regions of the village of Dongsui (river estuary town) (37°38′31.94”N, 118°51′41.69”E; altitude, 2 m) in Kenli county, Dongying, in northeast Shandong province, China.

Mosquitoes were collected from a pigsty at night using ultraviolet light traps (12 V; 300 mA; Wuhan Lucky Star Environmental Protection Tech Co., Ltd., Hubei, China). Traps were set from 7:00 pm to 7:00 am, which spanned the period from sunset to sunrise. The captured mosquitoes were frozen at − 20 °C for at least 40 min and identified based on morphology. The adult mosquitoes captured were identified according to morphological keys in Lu Baolin et al. [17, 18]. The mosquito species were selected on a chilled plate and only female mosquitoes were collected. Approximately 50–100 mosquitoes of the same species were pooled in cryogenic vials and stored in liquid nitrogen until further use [19].

### Virus isolation

Mosquitoes were removed from the liquid nitrogen and immediately homogenized and centrifuged as previously reported [20]. The supernatants were inoculated in a monolayer of C6/36 or BHK-21 cells in 24-well plates and incubated at 28 °C and 37 °C, respectively. Cells were observed for cytopathic effects (CPEs) every 8 h for 6–7 days after incubation for 24 h. A specimen was regarded as a positive isolate if it caused CPEs in three successive cell passages. Infected cell supernatants were stored at − 80 °C for further analysis.

### Virus electron microscopy

For negative staining, cell culture supernatants were absorbed on Formvar and carbon-coated grids for 1 min and stained with 1% (*w*/*v*) phosphotungstic acid (pH 6.8) for 1 min. Grids were air dried and observed under a Tecnai 12 transmission electron microscope (FEI, Eindhoven, Netherlands).

For ultrathin sections, cell pellets were fixed in a solution of 2% formaldehyde and 2.5% glutaraldehyde, post-fixed in 1% osmium tetroxide, dehydrated in an ethanol gradient, embedded in epoxy resin, and polymerized at 60 °C for 24 h. Ultrathin sections (80 nm) were obtained from the resin blocks, mounted on copper grids, and stained with uranyl acetate and lead citrate. Finally, the ultrathin sections were observed using transmission electron microscopy.

### Polyacrylamide gel electrophoresis (PAGE)

RNA was subject to PAGE on a discontinuous system with 5% acrylamide (acrylamide/bisacrylamide 29:1; Bio-Rad Laboratories, Hercules, CA, USA) stacking gel (with Tris-HCl buffer, pH 6.8) and 8% acrylamide resolving gel (with Tris-HCl buffer, pH 8.8). Following electrophoresis, the gel was stained with a Protein Silver Stain Kit (Leagene, Beijing, China) according to the manufacturer’s instructions and observed under white light. The KDV YN0557 was selected as positive control [13].

### Extraction of nucleic acids and RT-PCR assays for detection of virus RNA

Viral RNA was extracted from specimens with positive CPEs using the QIAamp Viral RNA Mini Kit (Qiagen, Valencia, CA, USA). Complementary DNA (cDNA) synthesis was performed using Ready-to-Go RT-PCR Beads (Amersham Biosciences Co., Piscataway, NJ, USA). Viral cDNA was subjected to PCR amplification with primers targeting the 12th segment of KDV [13] (KDV-12-F: 5’-GACGCTTTGAGATTATCTCGAC-3′, KDV-12-R: 5’-GCTCAATCGCATTCTCACC-3′) and GoTaqGreen Master Mix. PCR products were sequenced using BigDye Terminator chemistry (Applied Biosystems, Foster City, CA, USA).

### Deep sequencing

The library was prepared using NEBNext Ultra RNA Library Prep Kit for Illumina (New England Biolabs, Hitchin, UK). The library was quantified on Qubit 3.0 fluorometer and Agilent Bioanalyzer 2100. Paired-end sequencing (2 × 250 base pairs) was performed on an Illumina MiSeq v2 platform. For analysis, reads were filtered based on their length and mean quality values (Q30). KDV sequence reads were mapped against the published sequence. Mapping was performed using the Burrows-Wheeler aligner-MEM program [21]. The complete nucleotide sequences of the viruses reported in this study have been submitted to the GenBank database under accession numbers MG590140-MG590151.

### Phylogenetic analysis

Phylogenetic analysis was conducted based on the protein sequence deduced from the conserved RNA-dependent RNA polymerase gene of selected BAV, LNV and KDV. A multiple sequence alignment matrix was created using ClustalW software (http://clustalw.ddbj.nig.ac.jp/) with default settings. The aligned matrix data were confirmed manually, and the amino acid sequences were analyzed using the maximum-likelihood method. The statistical significance of the resulting tree was evaluated using a bootstrap test with 1000 replicates.

## Results

### Mosquito collection

On September 7, 2016, a total of 3000 mosquitoes representing two species, *Culex tritaeniorhynchus* (2300) and *Anopheles sinensis* (700) were collected in Kenli county, Shandong province, China.

### Virus isolation and identification

**Fig. 1:**
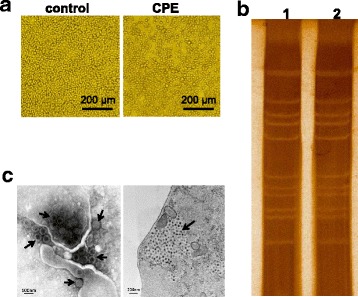
Virus isolation and identification. **a** Cytopathic effects (CPEs) caused by Kadipiro virus (KDV; SDKL1625) in C6/36 cells 72 h after inoculation. The non-infected control and infected cells are shown. **b** Electrophoretic migration patterns of the dsRNA of Kadipiro virus as determined by polyacrylamide gel electrophoresis. Lane 1: Positive control; lane 2: SDKL1625. **c** Electron micrograph of KDV shows virus particles with a diameter of approximately 70 nm. Arrows indicate KDV particles in infected cell culture supernatant; ultrathin sections show large, electron-dense viral inclusion body (arrow) within the cytoplasm composed of a large number of KDVs in infected C6/36 cells

The SDKL1625 of KDV was isolated from one (*Anopheles sinensis*) of 33 mosquito pools captured in Kenli county, Shandong province, China in 2016. The virus was shown to cause CPEs in C6/36 cells beginning at 72 h after inoculation in first-blind passage. The main characteristic of the CPEs was cell shedding, along with cell shrinking, or floating with eventual detachment from the growth surface (Fig. 1a). No CPEs were observed in BHK-21 cells during three blind passages.

RT-PCR assays were performed to detect the presence of the virus. Reactions with the KDV primers were positive with a product length of 370 bp. The YN0557 of KDV was isolated from mosquitoes in Yunnan province, China in 2009, and the RNA of this virus was used as a positive control in agarose gel electrophoresis [13]. After sequence analysis using the National Center for Biotechnology Information BLAST program (https://blast.ncbi.nlm.nih.gov/Blast.cgi), we preliminarily identified SDKL1625 as KDV.

The double-stranded RNA of the SDKL1625, which was purified and extracted from infected cell cultures, was analyzed by PAGE. PAGE analysis revealed that SDKL1625 exhibited the same pattern of bands as YN0557. The banding pattern consisted of 12 segments, forming a “6–5-1” pattern, which is an important characteristic of KDV (Fig. 1b).

### Virus electron microscopy

The KDV virus particles from the supernatant of infected C6/36 cells appeared spherical with a diameter of approximately 70 nm and the virion surface was covered with projections, presenting a clear double-layered capsid structure. Ultrathin sections showed electron-dense virus particles within the cytoplasm of KDV-infected C6/36 cells aggregated as viral inclusion bodies, which are thought to be the main site of replication, similar to the Banna virus (Fig. 1c) [22].

### Genome characterization

**Fig. 2:**
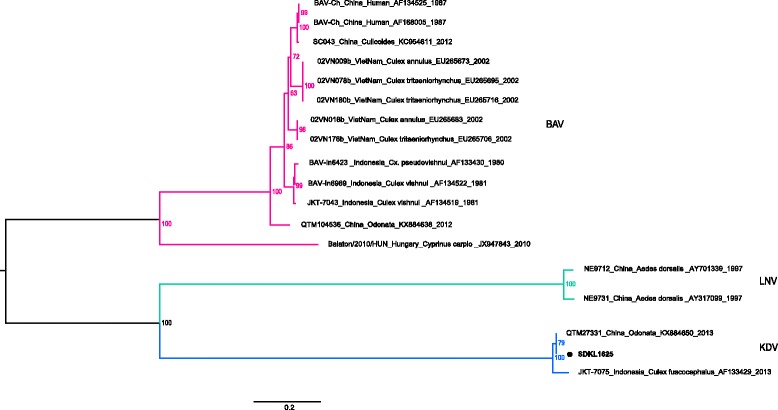
Molecular phylogenetic analyzed by the maximum-likelihood method. A maximum likelihood phylogenetic tree of Banna virus (BAV), KDV and Liao ning virus (LNV) was inferred based on the amino acid sequences of the first segments. BAV, LNV and KDV are labeled in red, green and blue, respectively. The newly discovered KDV strain is labeled with a solid circle. Numbers associated with branches indicate the percentage of 1000 bootstrap replicates that support the existence of these branches. Branches with < 60% bootstrap support have been collapsed

The complete genome sequences of SDKL1625 were characterized by next generation sequencing. A maximum-likelihood phylogenetic tree was inferred from the aligned amino acid sequences of segment 1 of the SDKL1625 together with two KDVs and 15 other representatives of the genus *Seadornavirus* (Fig. 2). In the phylogenetic tree, the SDKL1625 sequences clustered with the KDV sequences. Nucleotide and amino acid sequences from genome segments 1–12 of SDKL1625 were compared with homologous KDV sequences of JKT-7075 and QTM27331 (Table 1). Nucleotide sequence identities between SDKL1625 and JKT-7075 ranged from 81% (VP1 and VP3) to 91% (VP7 and VP9), and ranged from 86% (VP5) to 99% (VP1, VP3, VP6, VP7 and VP9) between SDKL1625 and QTM27331. The amino acid identities of SDKL1625 with the JKT-7075 and QTM27331 ranged from 86% (VP5) to 97% (VP2) and from 88% (VP5) to 99% (VP1, VP3, VP6, VP7 and VP9), respectively.

**Table 1:**
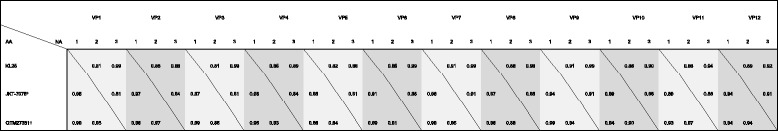
Sequence Identities Between discovered KDVs

* JKT-7075, isolated in central Java, in Bantul (Kadipiro), Yogyakarta, from Culex fuscocephalus mosquitoes in 1981 by J. D. Converse and was identified by Brown et al.

† QTM27331, isolated and identified in China, from Odonata in 2013 by Zhang,Y.Z. et al

## Discussion

KDV is a member of the genus *Seadornavirus*, family Reoviridae. There are three species of viruses in this genus: BAV, KDV and LNV. Each of these viruses has been isolated from *Aedes*, *Anopheles* and *Culex* mosquito populations, but only BAV has been shown to cause infection in humans [9, 23]. KDV has been isolated from *Culex*, *Anopheles*, *Armigeres* and *Aedes* mosquitoes in Indonesia, and from *Culex*, *Anopheles* and *Armigeres* mosquitoes in China [13]. This range includes the tropics and subtropics. Here, we isolated the SDKL1625 from *Anopheles sinensis* mosquitoes in Shandong province in eastern China. This is the first time KDV has been isolated from the northern temperate zone.

The genome of SDKL1625 shows variation compared with the previously discovered KDV isolates QTM27331 and JKT-7075. Phylogenetic analysis showed that SDKL1625 was more closely related to QTM27331 (Odonata, China, 2013) than to JKT-7075 (*Culex fuscocephalus*, Indonesia, 1981). SDKL1625 was isolated from *Anopheles sinensis* mosquitoes in China, which have similar origin but a greater genetic distance from JKT-7075. Indeed, SDKL1625 and QTM27331 collected in China have high degrees of sequence identity. Although QTM27331 was isolated from Odonata, we don’t consider Odonata as the natural host of KDV just because mosquitoes belong to the diet of the Odonata. Previous KDV isolates from Yunnan province were cultured with cell lines and characterized morphologically, however, the full genome was not sequenced [13]. The sequence data in this study represent the first complete genome sequence of KDV isolated in China. A recent study of a plasma sample from a febrile adult indicates that the tropism of KDV includes humans [16]. Thus, it will be important to conduct further investigation of KDV, and in particular, the association between KDV and human diseases.

Mosquito-borne viruses, such as BAV and LNV, were originally discovered in one location, but later, more extensive surveys provided a more accurate representation of the distributions of these viruses. BAV was first isolated in Yunnan province in 1987 [5], and isolates including three genera and 10 species have now been obtained from mosquitoes in various provinces of China (Gansu, Shanxi, Liaoning, Yunnan and Beijing) [8]. LNV was first isolated in Jilin province but additional isolates were later obtained from several regions in northern China [10]. KDV has now been isolated in both northern and southwestern China, suggesting that the distribution of KDV in China was originally underestimated.

This study described the isolation and characterization, as well as genomic analyses, of KDV SDKL1625 and its phylogenetic relationship with members of the genus *Seadornavirus*. These discoveries have enriched our knowledge of the distribution of KDV in China, and prompted further arbovirus investigation. Serologic screening should be conducted to determine the presence, prevalence and distribution of KDV.

## Conclusions

We describe the isolation, genome sequencing and characterization of the Kadipiro virus during arbovirus investigation in Shandong province. The genome of SDKL1625 revealed that the isolated virus was genetically close to strain isolated from Odonata in China. The sequence data in this study represent the first complete genome sequence of KDV isolated in China, which will further enrich our understanding of the molecular biological characteristics of KDV and seadornaviruses as well as the distribution of KDV in China.

## Abbreviations

BAV: Banna virus
CPE: cytopathic effect
KDV: Kadipiro virus
LNV: Liao ning virus
PAGE: Polyacrylamide gel electrophoresis
